# LncRNA MALAT1 as diagnostic and prognostic biomarker in colorectal cancers: A systematic review and meta-analysis

**DOI:** 10.1371/journal.pone.0308009

**Published:** 2024-10-29

**Authors:** Mahdi Masrour, Shaghayegh Khanmohammadi, Amirhossein Habibzadeh, Parisa Fallahtafti

**Affiliations:** 1 School of Medicine, Tehran University of Medical Sciences, Tehran, Iran; 2 Network of Immunity in Infection, Malignancy and Autoimmunity (NIIMA), Universal Scientific Education and Research Network (USERN), Tehran, Iran; 3 Non-Communicable Diseases Research Center, Endocrinology and Metabolism Population Sciences Institute, Tehran University of Medical Sciences, Tehran, Iran; 4 Research Center for Immunodeficiencies, Pediatrics Center of Excellence, Children’s Medical Center, Tehran University of Medical Sciences, Tehran, Iran; 5 Tehran Heart Center, Cardiovascular Diseases Research Institute, Tehran University of Medical Sciences, Tehran, Iran; Hawler Medical University, IRAQ

## Abstract

**Objective:**

This study investigated the relationship between the long non-coding RNA Metastasis-Associated Lung Adenocarcinoma Transcript 1 (MALAT1) expression and colorectal cancer (CRC) using a thorough systematic review and meta-analysis.

**Methods:**

Under the PRISMA guidelines, a systematic review was conducted on studies published from the databases’ inception to September 18, 2023. Prognostic value and diagnostic accuracy were explored. Additionally, the association between levels of MALAT1 expression and pathological features was investigated. The statistical analysis was performed using the “meta” package of R.

**Results:**

Among the pathological parameters examined, based on three studies involving 51 cases of metastatic CRC and 135 cases of non-metastatic CRC, a statistically significant correlation was found between the expression level of MALAT1 and distant metastasis, with an OR of 16.0118 (95% CI: 4.5618–56.2015). Three studies involving 378 cases reported overall survival and had a pooled HR of 2.3854 (95% CI: 1.3272–4.2875). Three studies involving 436 cases reported disease-free survival and had a pooled HR of 2.4772 (95% CI: 1.3774–4.4549). All prognosis studies utilized tumor tissue samples as specimens to assess the expression level of MALAT1. Case-to-control diagnostic studies with 126 cases and 126 controls had a pooled AUC value of 0.6173 (95% CI: 0.5436–0.6909), a pooled sensitivity of 0.675 (95% CI: 0.324–0.900), and a pooled specificity of 0.771 (95% CI: 0.685–0.839).

**Conclusions:**

The expression of MALAT1 in CRC is highly correlated with distant metastasis and has an impact on survival and prognosis. MALAT1 could also be employed as a diagnostic biomarker. More prospective studies should be performed to assess the MALAT1 diagnostic potential in the early stages of CRC.

## Introduction

Colorectal cancer (CRC) is one of the most frequent malignancies that affects millions of people worldwide despite significant advances in treatment. CRC accounts for 9.4% of all annual cancer-related deaths worldwide and is the second most deadly cancer worldwide [1]. Many factors play a crucial role in CRC development, including gene mutations, epigenetic changes, and local inflammatory changes. Several molecular pathways, including Wnt, KRAS, TP53, MLH1, and BRAF, are involved in the progression of CRC [2, 3].

It is essential to detect precancerous lesions and early-onset CRC in asymptomatic individuals at average risk [4]. Colonoscopy is consensually regarded as the gold standard for screening CRC due to its ability to detect and remove potentially precancerous colon polyps and diagnose CRC at an early stage [5]. Nevertheless, colonoscopy has challenges, including invasiveness and procedure-associated risks, demanding extensive bowel preparation, the dependence of results on the operator, and incurring high expenses, which make it less favorable [6]. Additionally, other screening methods, such as various serum biomarkers recently utilized for diagnosing or monitoring CRC progression, have limited sensitivity and specificity, which reduces their effectiveness for screening purposes [7]. Although treatments for CRC have improved significantly, the mortality and morbidity rates for this disease remain high [1]. When considering all aspects, it becomes apparent that there is a requirement for new therapeutic targets. Also, there is a need for new non-invasive biomarkers that can rapidly diagnose diseases, improve prognosis, and reduce the burden.

Recently, researchers studied biomarkers such as microRNAs (miRNAs) and long non-coding RNAs (lncRNAs) for non-invasive cancer diagnosis, assessment of prognosis, and treatment response evaluation [8, 9]. LncRNAs are a type of RNA molecule with a length of more than 200 nucleotides. LncRNAs represent a significant portion of non-coding RNAs and play vital roles in cell growth and tumorigenesis [10, 11]. They have shown promising results as biomarkers and can significantly improve diagnostic and prognostic accuracy for many types of cancer [12–14].

LncRNA Metastasis-Associated Lung Adenocarcinoma Transcript 1 (MALAT1) is a lncRNA that plays an important role in cellular proliferation, apoptosis, and migration [15]. In 2003, overexpression of MALAT1 was first discovered in metastatic tissue of non-small cell lung cancer patients [16]. According to various recent studies, there is a positive correlation between overexpression of MALAT1 and progression and metastasis in many other tumors such as breast cancer, ovarian cancer, prostate cancer, hematologic malignancies, sarcoma, bladder cancer, gastric cancer, hepatocellular carcinoma, esophageal squamous cell carcinoma, renal cell carcinoma and CRC [15, 17–20]. Therefore, we decided to analyze MALAT1 because of its significant role in the development of different types of tumors and its potential diagnostic and prognostic value in CRC, as demonstrated in the various studies.

MALAT1 can influence cancer development by activating Wnt/-catenin, ERK/MAPK, and PI3K/AKT signaling pathways. This lncRNA promotes tumor angiogenesis, significantly increasing cancer metastasis, is involved in tumor immunity, and is associated with chemoresistance and radiation resistance of several cancers [21]. Recent studies found that MALAT1 plays a vital role in CRC pathogenesis by targeting multiple signaling pathways and miRNAs. This marker can potentially predict and diagnose cancer [20, 22]. However, the clinical application of MALAT1 in CRC has not been widely considered, and the limitations of performed studies with small sample sizes make their results different and unreliable. Our study aims to provide an evidence-based medical reference by meta-analyzing the diagnostic and prognostic value of lncRNA MALAT1 expression in CRC.

Taken together, these data suggest a potential association between MALAT1 expression levels and early diagnosis, prognosis, and treatment outcomes in CRC patients. Therefore, we conducted this systematic review and meta-analysis to uncover the diagnostic and prognostic significance of MALAT1 in CRC patients and analyze its association with the clinical and histopathological features of this disease.

## Methods

A systematic review and meta-analysis were conducted in adherence to the PRISMA guidelines [23]. The protocol for our systematic review and meta-analysis has been officially registered at PROSPERO. The registration number assigned to our study is CRD42023476807.

### Literature search

A comprehensive search was conducted in the PubMed, Web of Science (ISI), Scopus, and Embase databases to identify English publications. We considered studies published from the databases’ inception to September 18, 2023, for inclusion without imposing any restrictions on the publication year. The databases were queried using a combination of Medical Subject Headings (MeSH) terms and free-text keywords: “MALAT1” and “colorectal cancer” and their expansions. The **S1 File** provide the search query.

### Inclusion and exclusion criteria

This review considered papers eligible if they met the following criteria: 1) they were original peer-reviewed papers, 2) utilized samples from patients who had been pathologically diagnosed with CRC, and 3) reported sensitivity, specificity, or area under the curve (AUC) values for MALAT1 in diagnosing CRC in a case-control study design, or 4) reported the association between MALAT1 expression and prognosis in terms of overall survival (OS), progression-free survival (PFS), disease-free survival (DFS), recurrence-free survival (RFS), and event-free survival (EFS) in a retrospective or prospective cohort study design, or 5) categorized patients based on levels of MALAT1 expression and pathological features such as tumor stage, tumor tissue differentiation, lymph node metastasis status, and distant metastasis.

Papers were deemed ineligible and thus excluded from the analysis if they met the following criteria: 1) they were non-English studies, 2) they were datasets, 4) studies that utilized animal models, 3) letters, comments, reviews, editorials, and conference abstracts, and 6) case reports and case series. There were no eligibility restrictions based on the healthcare settings in which the research was conducted, nor were there any eligibility restrictions based on the total number of participants in the included studies.

### Study selection and data extraction

After removing any duplicates, SK and PF evaluated the papers’ eligibility in accordance with the inclusion and exclusion criteria previously defined. After constructing a list of studies that met the eligibility criteria, both authors independently reviewed the full texts. Conflicts that arose during the review process were effectively resolved through the formulation of a consensus.

Two investigators (PF and MM) independently collected data from the included studies in an electronic spreadsheet. Author, publication year, study design, specimen type, sample size, control population, MALAT1 expression levels in patients relative to the control group, diagnostic or prognostic performance measures, including sensitivity, specificity, AUC with corresponding 95% confidence interval (CI) and p-value, as well as mean, median, and hazard ratio (HR) for OS, PFS, DFS, EFS, and RFS were extracted from each study when available. Additionally, data was collected regarding the number of patients classified according to the levels of MALAT1 expression in various clinical and pathological parameters, including pathological tumor staging, tumor tissue differentiation level, lymph node metastasis status, and distant metastasis status in a separate spreadsheet. The patients were categorized into high or low expression levels in the studies, depending on their MALAT1 expression relative to the median expression of each study. Disagreements were resolved through dialogue and agreement.

### Quality assessment

The Newcastle-Ottawa Scale (NOS) was used to evaluate the quality of cohort and case-control studies included in the analysis. This scale provides a numerical evaluation of the research quality. It uses a star system to assess each study based on eight criteria that fall into three primary categories: selection of the study groups, comparability of the groups, and ascertainment of either the exposure or outcome of interest, using a star system. When interpreting the scores, studies were deemed to have ‘good quality’ if they received seven or more stars. Studies were classified as ‘fair quality’ if they received 5 or 6 stars and “poor quality” if they received less than 5 stars [24]. Two investigators (PF and MM) evaluated the quality of each study independently based on predetermined criteria. Discrepancies in quality evaluation were resolved through communication or consultation with an additional reviewer.

### Statistical analysis

The statistical analysis and visualizations were performed using the “meta” package of R version 4.2.2 (R Core Team [2021], Vienna, Austria) [25]. We conducted a meta-analysis on the AUC data using the “metagen” function of the “meta” package in R. To address the expected heterogeneity among studies, we utilized the random effects model with the inverse variance method, which assigns studies a weight inversely proportional to their variance for each random variable. For the prognostic data, after calculating logarithmic HRs, a meta-analysis was also performed using the random effects model with the inverse variance method using the “metagen” function. We calculated the odds ratios (OR) based on the provided data to evaluate the association between high or low MALAT1 expression and clinical or pathological characteristics. Subsequently, we conducted a meta-analysis on the ORs using the random effects model and the inverse variance method, employing the “metagen” function. In the staging section, a comparison was made between higher stages (III and IV) and lower stages (I and II). In the section on tissue differentiation, poor differentiation was compared to well/moderate differentiation. In the sections pertaining to lymph node metastasis and distant metastasis, the occurrence of metastasis was compared to the absence of metastasis. In both high- and low-expression groups, the term “events” refers to the count of individuals exhibiting certain pathological characteristics. The Bonferroni correction method was employed to adjust the p-values of the meta-analysis results, as these data were collected from the same group of individuals in the pathological characteristics section [26].

The 95% CI was used to calculate the standard error of the AUCs for use in meta-analysis. If CI was not provided, the Hanley and McNeil method was used to calculate the standard error from the AUC value and sample size [27, 28]. The study employed I2 and tau2 statistics to assess heterogeneity. Statistical significance was determined by an I2 value exceeding 50% and a p-value below 0.05.

## Results

### Basic characteristics

**Fig 1:**
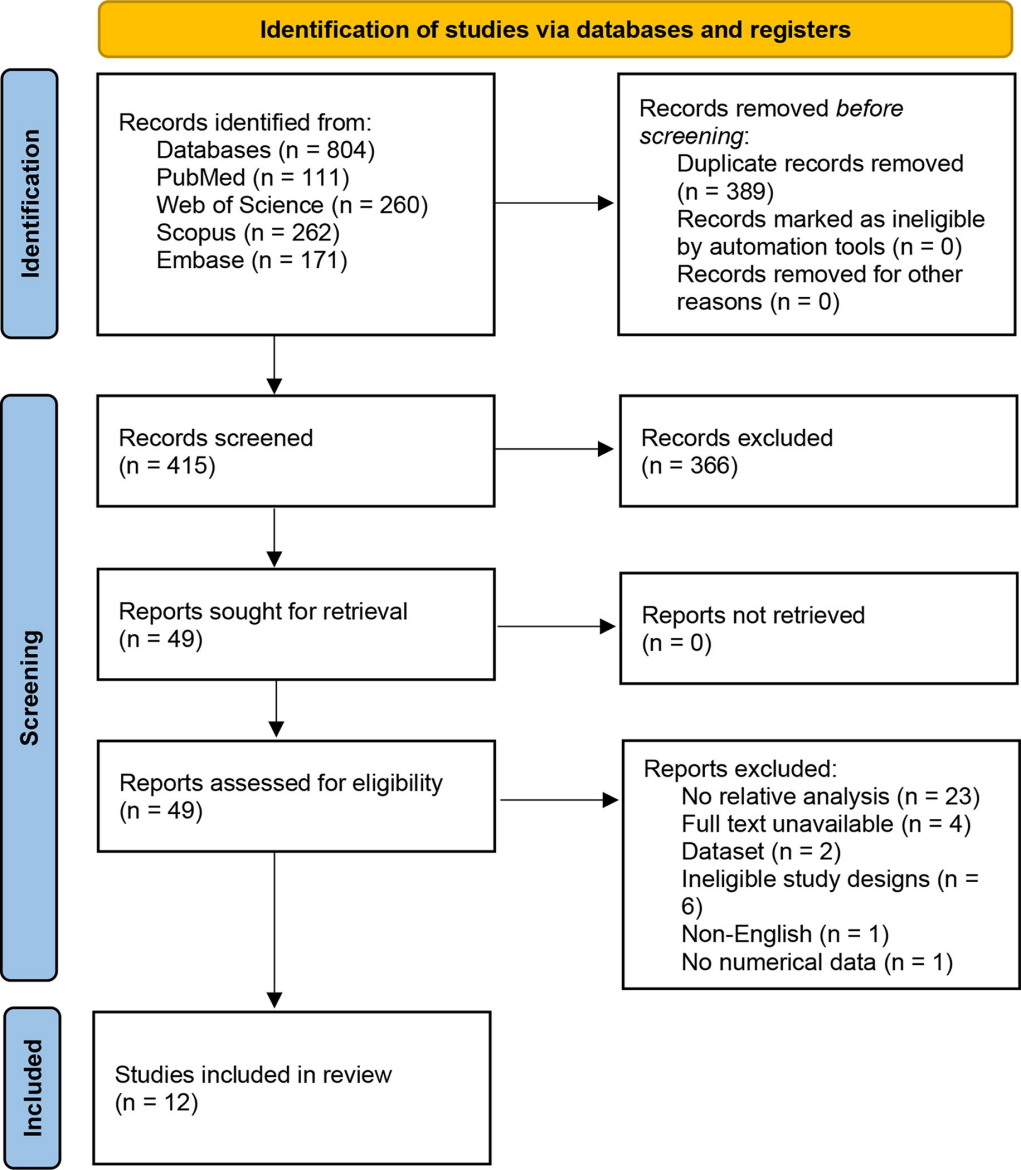
The PRISMA flowchart. Illustrating the process of selecting the studies. The diagram depicts the number of records that were identified, included, and excluded, as well as the reasons for exclusions.

After executing database searches, 804 titles were identified. After removing duplicates, 415 papers remained for further evaluation and screening. Following titles and abstracts screening, 366 studies were excluded, and 49 papers were deemed suitable for full-text review. Finally, three studies met the requirements for inclusion in the diagnostic accuracy section of this review, while four studies met the criteria for inclusion in the prognosis and survival section. Additionally, nine studies were included in the pathological and clinical section. The **S1 File** include a list of the excluded studies. The procedure for choosing and excluding studies is described in the PRISMA flowchart, which is shown in **Fig 1**.

A brief summary of the main characteristics of the studies that were included is given in **Table 1**. The prognosis section’s papers were released between 2014 and 2022, whilst the diagnosis section’s articles were published between 2019 and 2021. The prognosis meta-analysis section analyzed a total of 378 CRC cases from China and Turkey to assess OS. Additionally, 436 cases from China and Turkey were examined to evaluate DFS. Tumor tissue was used for two diagnostic studies [29, 30], while fecal samples were used in only one study [31]. All prognosis assessments were on tumor tissue samples.

**Table 1:** Basic characteristics of included studies.

ID	Author, year	Country	Specimen	Control type	Case No.	Control No.	Regulation	Histopathological parameter		No. of participants MALAT1		Sensitivity	Specificity	AUC (95% CI)	p-value	Overall survival (95% CI)	p-value	Disease-free survival (95% CI)	p-value
										Low	High								
1	Chaleshi, 2020 [29]	Iran	CRC tissue	healthy adjacent tissues	66	66	Up	Grade	I	6	4	0.5	0.75	0.603 (0.706–0.501)	<0.05				
									II	5	7								
									III	4	0								
								Stage	I	6	4								
									II	1	3								
									III	8	4								
								Tumor Size	<5 cm	3	5								
									≥5 cm	12	6								
2	Huang, 2020 [69]	China	CRC tissue		50		Up	Stage	I	8	2								
									II	8	5								
									III	16	4								
									IV	7	1								
								Lymph node metastasis	No	19	8								
									Yes	18	5								
3	Li, 2022 [37]	China	CRC tissue		164		Up	AJCC Stage	I	10	6					1.428 (2.263–0.901)	0.13	1.525 (2.387–0.975)	0.065
									II	26	40								
									III	35	31								
									IV	11	5								
								Tumor Size	≤ 40	31	27								
									> 40	51	55								
								Histopathological Morphology	Protruding	60	54								
									Infiltrating ulcer	22	28								
								Tumor differentiation	Low to Medium	52	51								
									High	30	31								
4	Li, 2017 [36]	China	CRC tissue		68		Up	Stage	I	16	5					2.9 (4.6–1.83)	<0.0001		
									II										
									III	18	29								
									IV										
								pT	pT1+pT2	18	7								
									pT3+pT4	16	27								
								pN	pN0	16	7								
									pN1	13	15								
									pN2	5	12								
									pN1+pN2	18	27								
								pM	pM0	32	24								
									pM1	2	10								
								Tumor differentiation	Well	8	6								
									Moderate	17	16								
									Poor	9	12								
5	Li, 2016 [70]	China	CRC tissue		30		Up	TMN Stage	I										
									II	9	11								
									III	6	4								
									IV										
6	Qiu, 2016 [71]	China	CRC tissue		120		Up	Stage	I										
									II	28	18								
									III	32	42								
								Tumor Size	small	29	22								
									big	31	38								
								Poor differentiation grade	well/moderate	36	31								
									poor	24	29								
								Lymph vascular invasion	Absence	31	41								
									Presence	29	19								
								Perineural invasion	Absence	33	46								
									Presence	27	14								
7	Xiong, 2018 [34]	China	CRC tissue		58		Up	Tumor differentiation	well	7	6								
									moderate	14	13								
									poor	8	10								
								Local invasion	T1+T2	16	6								
									T3+T4	13	23								
								Lymph node metastasis	N0	14	6								
									N1+N2	16	22								
								Distant metastasis	M0	29	20								
									M1	1	8								
8	Zheng, 2014 [38]	China	CRC tissue		146		Up	Tumor differentiation	Well/moderate	57	56					3.968 (9.456–1.665)	0.002	2.863 (4.943–1.659)	<0.001
									Poor	16	17								
								T stage	T2/3	25	25								
									T4	48	48								
								N stage	N0	24	31								
									N1/2	49	42								
								TNM stage	II	24	31								
									III	49	42								
								Lymph vascular invasion	Absence	50	54								
									Presence	23	19								
								Perineural invasion	Absence	54	60								
									Presence	19	13								
9	Zhang, 2020 [35]	China	CRC tissue		60		Up	Tumor differentiation	High	10	10								
									Moderate	11	15								
									low	7	8								
								Metastasis	Yes	3	27								
									No	25	5								
								Stage	I+II	18	12								
									III+IV	10	20								
10	Gharib, 2021 [31]	Iran	Fecal samples	normal	60	60	Up					0.8182	0.7778	0.6331 (0.7413–0.525)	4.4 E−05				
11	Ji, 2019 [30]	China	CRC tissues with matched hepatic or lung metastasis	CRC tissues without metastasis	46	78	Up							0.8673					
12	Ak Aksoy, 2022 [39]	Turkey	CRC tissue		126		Up											4.325 (9.977–1.875)	0.0009

AJCC, American Joint Committee on Cancer; AUC, area under the curve; CI, confidence interval; CRC, colorectal cancer; TNM staging, tumor, node and metastasis staging

### Quality assessment

Independent investigators evaluated the quality of the included studies using the NOS (**Table 2**). There was little chance of bias for the included studies as 10 of them, or 83.3% of the total, had a “good” score, 2 of them, or 16.7% of the total, had a “fair” score, and no study had a “poor” score.

**Table 2:** The Newcastle-Ottawa Scale quality assessment.

Author, year	Selection				Comparability	Exposure			Overall score
	Case definition	Representativeness	Selection of Controls	Definition of Controls		Ascertainment of exposure	Same method of ascertainment	Non-Response rate	
Zheng H., 2014 [38]	*	*			**	*	*	*	7
Qiu Gu., 2016 [71]	*	*	*	*	**	*	*	*	9
Xiong Y., 2018 [34]	*		*		**		*	*	6
Zhang J., 2020 [35]	*	*	*	*	**	*	*	*	9
Chaleshi V., 2020 [29]	*	*			**	*	*	*	7
Huang B., 2020 [69]	*	*			**	*	*	*	7
Li H., 2022 [37]	*				**	*	*	*	6
Li P., 2017 [36]	*	*			**	*	*	*	7
Li Q., 2016 [70]	*	*			**	*	*	*	7
Gharib E., 2021 [31]	*	*	*	*	**	*	*	*	9
Ji Q., 2019 [30]	*	*	*	*	**	*	*	*	9
Ak Aksoy S., 2022 [39]	*	*			**	*	*	*	7

### Meta-analysis of diagnostic value of MALAT1 in CRC

**Fig 2:**
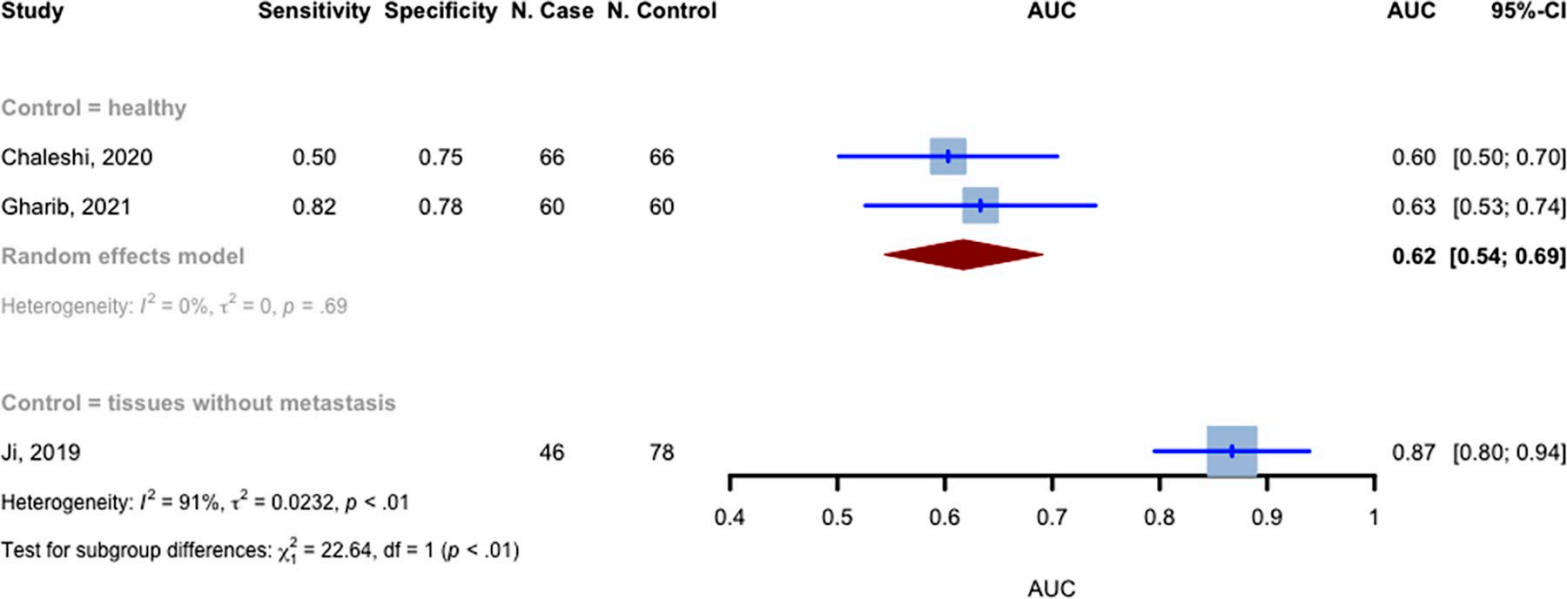
The AUCs meta-analysis. Illustrating the findings of the meta-analysis of the area under the curve (AUC) values. The studies included in the analysis were grouped based on the type of cases and controls. The random effects model was used, along with the inverse variance method, to calculate the pooled AUC value and its corresponding 95% confidence interval (CI). The heterogeneity among the studies was assessed using the I^2 and Tau^2 measures.

The AUC values for MALAT1 on the diagnosis of CRC were published in three of the included studies [29–31]. Ji et al. [30] compared tissue samples with matched lung or liver metastases to tissues without metastases, while Chaleshi et al. [29] and Gharib et al. [31] compared samples from cancer patients with non-cancerous samples. For case-to-control studies with 126 cases and 126 controls, the random effects model with inverse variance method produced a pooled AUC value of 0.6173 (95% CI: 0.5436–0.6909) (**Fig 2**). The pooled AUC value of 0.6173 indicates that the MALAT1 model for diagnosis has a 61.73% chance of correctly distinguishing between a randomly chosen positive case (a patient with CRC) and a negative case (a healthy control), assigning a higher risk score to the former. By comparing these findings to other biomarkers used for CRC, additional insights can be gained. For example, certain studies have discovered that the sensitivity, specificity, and AUC value of individual extracellular vesicle (EV) RNAs and EV RNA panels for early CRC detection were 76%, 75%, and 0.87, and 82%, 79%, and 0.90, respectively [32]. Furthermore, additional research has indicated that metabolites, specifically palmitoylcarnitine and sphingosine, have the potential to be used as biomarkers. These biomarkers have demonstrated AUC values exceeding 0.80 in both serum and cells [33].

All three studies demonstrated statistically significant up-regulation of MALAT1 expression levels in CRC. Furthermore, Chaleshi et al. [29] noted a sensitivity and specificity of 0.5 and 0.75, respectively. Gharib et al. [27] reported that the corresponding values for specificity and sensitivity were 0.7778 and 0.8182, respectively. For the aforementioned studies, which included 126 cases and 126 healthy controls, the pooled sensitivity was 0.675 (95% CI: 0.324–0.900, p = 0.328), and the pooled specificity was 0.771 (95% CI: 0.685–0.839, p < 0.0001). For differentiating metastatic from non-metastatic CRC, MALAT1’s AUC value was 0.8673 (95% CI: 0.7953–0.9393), as reported by Ji et al. [30], involving 46 metastatic cases and 78 non-metastatic controls.

A statistical test was conducted to examine subgroup differences between the two groups of AUC values. The results indicated a statistically significant difference (p < 0.0001) between two studies that compared CRC with healthy controls and one study that compared metastatic with non-metastatic controls. This finding suggests that MALAT1 plays a more prominent role in the formation of metastasis rather than cancer formation.

### Meta-analysis of clinical and histopathological characteristics

**Fig 3:**
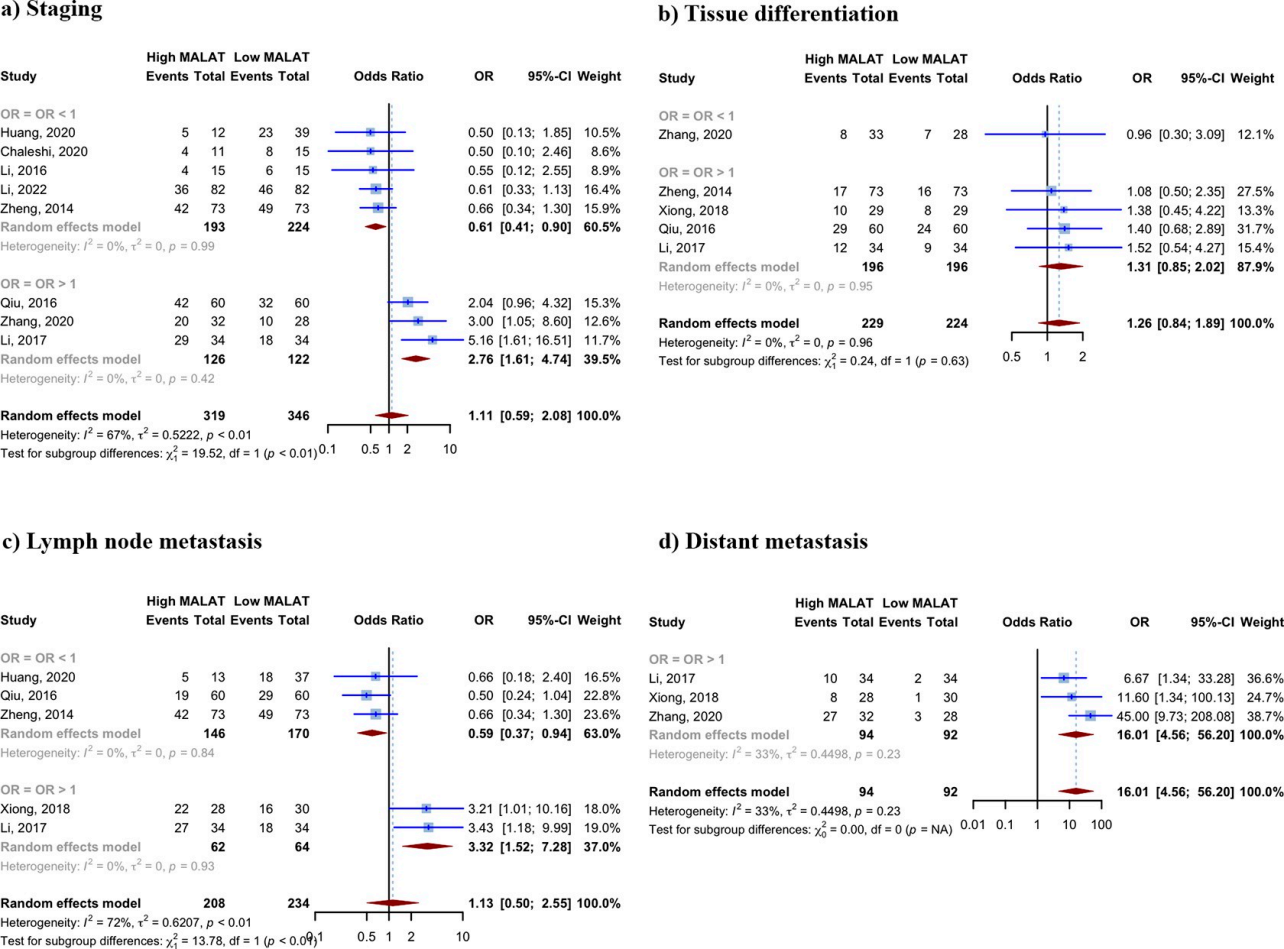
Meta-analysis of histopathological characteristics. Illustrating the findings of the meta-analysis of the odds ratios (ORs) calculated for the correlation between colorectal cancer pathological characteristics and MALAT1 expression. For better representation, the studies are categorized as OR >1 and <1. The random effects model was used, along with the inverse variance method, to calculate the pooled OR and its corresponding 95% confidence interval (CI). The heterogeneity among the studies was assessed using the I^2 and Tau^2 measures.

Nine studies have provided information on the patient population in high and low MALAT1 expression groups, with the classification based on clinical and pathological characteristics. The summary of findings is presented in **Table 3**, and forest plots are illustrated in **Fig 3**. In forest plots, the studies are categorized into subgroups based on whether their OR is higher or lower than one.

**Table 3:** Summary of findings for meta-analysis of colorectal cancer histopathological characteristics based on MALAT1 expression.

Pathological parameter	Number of studies	Estimated OR [95% CI]		Heterogeneity I2	Bonferroni corrected the p-value (original p-value)	Studies with OR < 1: Studies with OR > 1	p-value for subgroup differences
**Staging (stages III/IV vs. I/II)**	8	1.1109	[0.5939; 2.0780]	67.4%	1 (0.7421)	5:3	< 0.0001
**Tissue differentiation (poor differentiation vs. well/moderate)**	5	1.2596	[0.8384; 1.8924]	0.0%	1 (0.2664)	1:4	0.6270
**Lymph node metastasis (metastatic vs. non-metastatic)**	5	1.1256	[0.4965; 2.5520]	71.7%	1 (0.7769)	3:2	0.0002
**Distant metastasis (metastatic vs. non-metastatic)**	3	16.0118	[4.5618; 56.2015]	32.7%	< 0.0004 (< 0.0001)	0:3	All studies OR > 1

CI, confidence interval; OR, odds.

Among the four pathological parameters examined, based on three studies [34–36] involving 51 cases of metastatic CRC and 135 cases of non-metastatic CRC, a statistically significant correlation was found between the expression level of MALAT1 and distant metastasis, with an OR of 16.0118 (95% CI: 4.5618–56.2015, p < 0.0001; I^2 = 32.7%). No statistically significant correlation was found between the expression level of MALAT1 and any of the three other pathological parameters that were examined, including tumor staging, lymph node metastasis, and tissue differentiation.

### Meta-analysis of the prognostic value of MALAT1 in CRC

**Fig 4:**
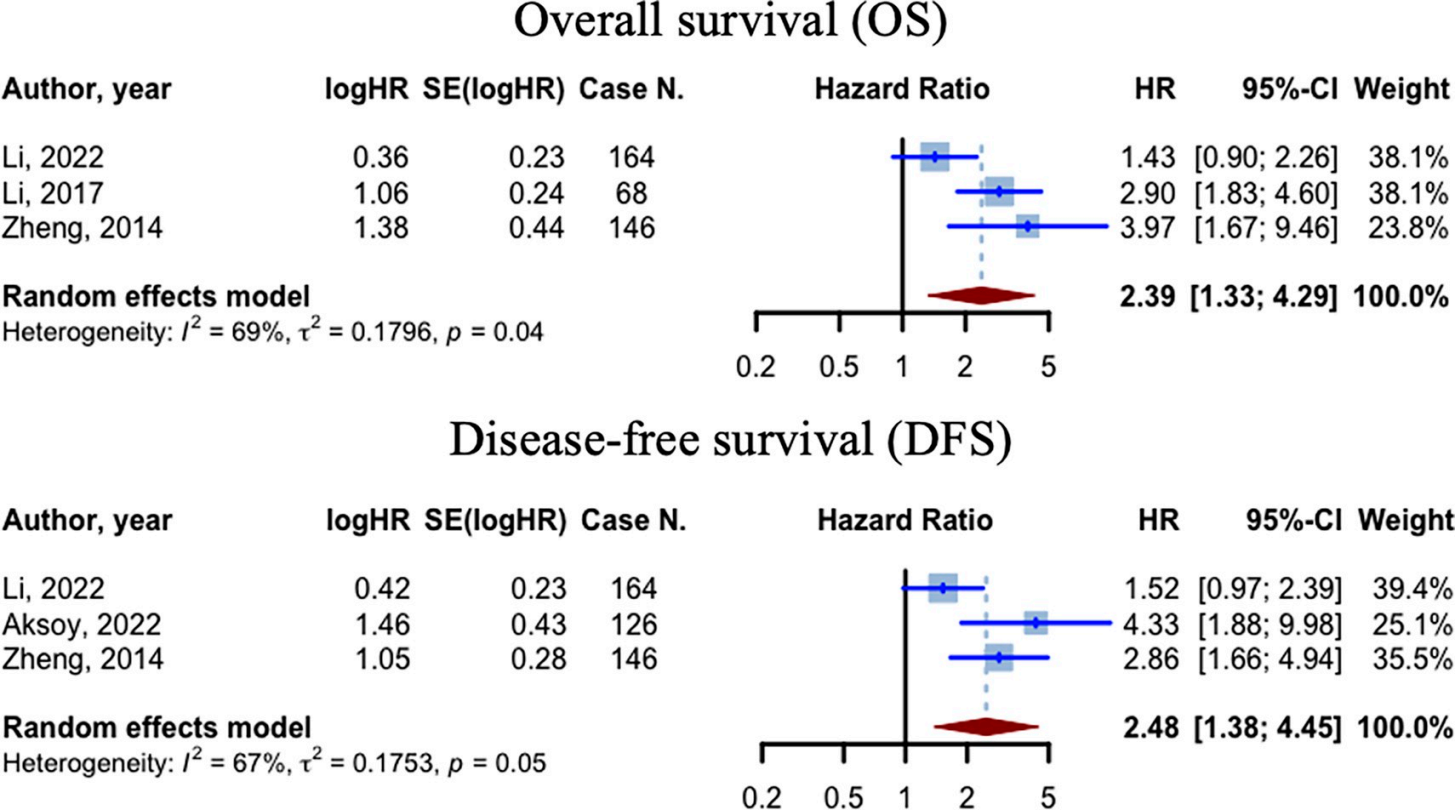
The overall survival and disease-free survival hazard ratios’ meta-analysis. Illustrating the findings of the meta-analysis of the survival outcomes represented by hazard ratios (HRs). The random effects model was used, along with the inverse variance method, to calculate the pooled HR and its corresponding 95% confidence interval (CI). The heterogeneity among the studies was assessed using the I^2 and Tau^2 measures.

All three studies [36–38] evaluating OS had reported HRs greater than 1. The cumulative HR for these studies was 2.3854 (95% CI: 1.3272–4.2875, p = 0.0037; I^2 = 69.4%). The aforementioned pooled HR value is derived from the evaluation of 378 cases of CRC. All three studies utilized tumor tissue samples as specimens to assess the expression level of MALAT1 (**Fig 4**).

Moreover, all three included studies [37–39] evaluating DFS had reported HRs greater than 1. The cumulative HR for these studies was 2.4772 (95% CI: 1.3774–4.4549, p = 0.0025; I^2 = 66.6%). The aforementioned pooled HR value is derived from the evaluation of 436 cases of CRC. All three studies utilized tumor tissue samples as specimens to assess the expression level of MALAT1 (**Fig 4**).

## Discussion

We meticulously performed a systematic review and meta-analysis of studies exploring the role of lncRNA MALAT1 in patients afflicted with CRC. Our objective was to elucidate the diagnostic accuracy of lncRNA MALAT1 and the correlation between its expression levels and disease prognosis in patients with CRC. We also explored correlations between MALAT1 expression and clinical characteristics. Based on our analysis, it has been observed that MALAT1 exhibits a more significant role in metastasis formation compared to other characteristics associated with CRC. The examination of clinical and histopathological characteristics revealed a notable association between the expression of MALAT1 and distant metastasis, with an OR of 16.0118 (95% CI: 4.5618–56.2015, p < 0.0001). The results also indicated that increased expression of MALAT1 is linked to unfavorable prognosis. The cumulative HR for OS and DFS in the meta-analysis of 378 cases and 436 cases, respectively, were 2.3854 (95% CI: 1.3272–4.2875, p = 0.0037) and 2.4772 (95% CI: 1.3774–4.4549, p = 0.0025). The meta-analysis of diagnostic values also revealed a pooled sensitivity of 0.675 and a specificity of 0.771 for up-regulation of MALAT1.

Biomarkers are molecular patterns that serve as helpful tools in early cancer detection and individualized treatment for CRC patients [40]. Early diagnosis in asymptomatic patients remains a pivotal objective for achieving positive survival outcomes. This entails the identification of early CRC and pre-malignant lesions, such as high-risk polyps. Moreover, prognostic biomarkers hold the potential to predict the advancement of diseases, encompassing the onset of recurrence and mortality, even in their early stages [41].

Recent advances have shown that miRNAs and lncRNAs are potential biomarkers in CRC [42]. LncRNAs participate in numerous biological functions, including regulation of transcription, RNA splicing, protein transportation and stability, cellular metabolism, and epigenetic regulation [43–46]. LncRNAs have been extensively studied in CRC and have been found to play significant roles in various aspects of CRC development and progression [36, 38, 40–42]. They are significantly implicated in CRC pathogenesis by acting as oncogenes or tumor suppressor genes [47]. For instance, maternally expressed gene 3 functions as a tumor suppressor, whilst HOX transcript antisense intergenic RNA and MALAT1 have significant oncogenic impacts [44, 48, 49]. The dysregulation of lncRNAs in CRC highlights their potential as diagnostic markers and therapeutic targets for this disease. Moreover, researchers investigate using lncRNA-based therapies, such as antisense oligonucleotides (ASOs), to target specific lncRNAs involved in CRC [50]. By inhibiting or silencing lncRNAs, it may be possible to disrupt their oncogenic functions and inhibit tumor growth [51, 52].

LncRNA MALAT1 has been extensively studied in various cancers, including CRC. It has different known functions and actions in numerous cellular processes, including cell proliferation, cell death, cell migration, and invasion [21]. Particularly, it interacts with proteins involved in cell cycle regulation, such as cyclins and cyclin-dependent kinases, to promote cell division and proliferation [53]. MALAT1 plays a significant role in cell death processes through apoptosis regulation, autophagy inhibition, and modulation of pyroptosis [54–56]. MALAT1 also promotes cell migration and invasion, which are crucial steps in cancer metastasis. It modulates the expression of genes involved in cell adhesion, extracellular matrix remodeling, and cytoskeletal dynamics, facilitating cancer cell movement and invasion into surrounding tissues [57, 58]. Generally, It is important to note that the role of MALAT1 in cellular processes can vary depending on the specific cellular context and cancer type [21].

MALAT1 has been found to interact with A-kinase anchor protein 9 (AKAP-9) in CRC cells [46]. AKAP-9 is a multivalent scaffold protein that is involved in organizing signaling complexes and regulating cellular processes [59]. Previous studies have shown that MALAT1 enhances AKAP-9 expression in CRC cells *in vitro*, functionally promoting cancer cell proliferation, invasion, migration, and metastatic spread [60, 61].

In our meta-analysis, we discovered a statistically significant correlation between the expression level of MALAT1 and distant metastasis. Furthermore, overexpression of MALAT1 was associated with poor OS and DFS, indicating that the expression level of MALAT1 is a potential prognostic tool for CRC patients. Studies of cancer tissue have shown that the increased expression of MALAT1 is associated with poorer prognosis in CRC patients [38, 62]. MALAT1 promotes CRC cell proliferation, invasion, and migration via up-regulating sex-determining region Y-box 9 in CRC cells [63]. Furthermore, silencing MALAT1 has been observed to increase the expression of microRNA-184 (miR-184) when activating Caspase3 activity, as well as inhibiting the expression of Bcl-2 and increasing the expression of Bax proteins; this indicates that miR-184 is the target miRNA of Lnc-RNA MALAT1 and MALAT1 induces CRC cell progression via inhibition of miR-184 [64]. MALAT1 also promotes CRC metastasis by increasing the transcriptional level of proto-oncogene Runt-related transcription factor 2 through the LRP6-mediated β-catenin signaling pathway. High MALAT1 expression has been significantly linked to an increased risk of tumor recurrence after surgical resection [30]. Additionally, MALAT1 overexpression is associated with chemoresistance and radioresistance, leading to poorer treatment outcomes and prognosis [36, 62].

MALAT1 may be a promising therapeutic target for the treatment of CRC. For instance, targeting MALAT1 by CRISPR/Cas9 may have therapeutic potential applications in CRC, particularly in the treatment of metastatic disease. CRISPR/Cas9-mediated inhibition of MALAT1 may inhibit cell proliferation and migration and induce apoptosis, thus improving patient prognosis. Recently, several studies have explored various strategies to target MALAT1 or its related pathways for potential therapeutic interventions [65]. ASOs can potentially antagonize MALAT1 and promote its degradation. ASOs are short synthetic DNA or RNA-based structures that can specifically bind to target RNA sequences, including MALAT1 [66]. Studies also revealed that small interfering RNA (siRNA) molecules can be designed to specifically target and silence the expression of MALAT1 [65]. Introducing siRNA molecules into cancer cells reduced the levels of MALAT1, and MALAT1 inhibition significantly modulated chemokine (C-C motif) ligand 5-induced migration and invasion of CRC cells [67]. Additionally, siRNAs restored oxaliplatin sensitivity in oxaliplatin-resistant CRC patients through targeted inhibition of both MALAT1 and enhancer of zeste homolog 2 [36].

The inhibition of MALAT1 significantly reduces tumor growth, invasion, and metastasis in CRC *in vitro* and *in vivo* [68]. Additionally, studies discovered that CRC patients with lower MALAT1 expression levels in primary tumors had better treatment outcomes and longer OS [30]. The downregulation of MALAT1 enhanced the sensitivity of the human colorectal carcinoma cell line HCT-116 to 5-fluorouracil by targeting miR-20b-5p [68]. In addition, high MALAT1 expression is associated with inadequate response to oxaliplatin-based chemotherapy by suppressing E-cadherin signaling and promoting epithelial-mesenchymal transition in CRC patients [36, 65]. Therefore, MALAT1 may be a potential therapeutic target in these patients. Furthermore, findings related to MALAT1-induced radioresistance elucidated that MALAT1 could be a promising therapeutic target for CRC patients with radioresistance [62]. Then, MALAT1 has the potential to develop personalized treatment approaches and combination therapy in CRC. For instance, it may serve as a valuable tool for detecting patients who are more likely to respond positively to specific therapeutic interventions.

Our study had several limitations that need to be mentioned. Firstly, there was no consensus among the included studies on specific cutoff values for high and low MALAT1 expression. Some of the included studies had retrospective designs, which could lead to bias in our results. Studies had discrepancies in methods and analyses, such as different sample sizes, sample types, outcome measures, and follow-up periods, resulting in heterogeneity. The results of small studies with limited sample sizes are likely to be biased. Also, most of the included studies have been conducted on Asian populations, especially Chinese populations. Thus, conducting more studies involving other countries and ethnicities is necessary to ensure the generalizability of our results.

While MALAT1 shows promise as a diagnostic and prognostic biomarker and also a therapeutic target for CRC, further large, independent cohorts are needed to validate these findings and establish its reproducibility and generalizability as a biomarker. Due to the methodological challenges encountered, establishing standardized protocols and more rigorous research on MALAT1 as a diagnostic and prognostic biomarker for CRC is necessary. Furthermore, different molecular subtypes of CRC have distinct characteristics and clinical behaviors. Future studies should focus on analyzing MALAT1’s role within specific molecular subtypes of CRC.

In summary, our research demonstrated that MALAT1 shows promise as a reliable prognostic biomarker for CRC patients, with possible implications for practical clinical use. Our results suggest that MALAT1 may be significantly involved in distant metastasis in CRC. We found a significant correlation between MALAT1 expression levels and distant metastasis but no such correlation with other pathological factors such as tumor staging, lymph node metastasis, and tissue differentiation. High MALAT1 expression was significantly linked to poorer OS and DFS, indicating its potential as a prognostic tool. The involvement of MALAT1 in CRC is noteworthy. Further investigation is needed to assess its potential in treatment strategies and improving patient outcomes in CRC clinical management.

## Supporting information

S1 File Search strategy and table of exclusion. (DOCX)

S2 File PRISMA checklist. (DOCX)
