# Supplementary material for: LncRNA MALAT1 as diagnostic and prognostic biomarker in colorectal cancers: A systematic review and meta-analysis
Source: PLoS One. 2024 Oct 29;19(10):e0308009. doi: 10.1371/journal.pone.0308009 (PMC11521308; doi:10.1371/journal.pone.0308009)
Supplement: S1 File — (DOCX) [file pone.0308009.s001.docx]

**Search strategy:**

PubMed:

(“MALAT1”[tiab] OR “MALAT 1”[tiab] OR “MALAT-1”[tiab] OR “metastasis associated lung adenocarcinoma transcript 1”[tiab] OR “metastasis associated lung adenocarcinoma transcript-1”[tiab] OR “NEAT2”[tiab] OR “NEAT 2”[tiab] OR “NEAT-2”[tiab] OR “non-coding nuclear-enriched abundant transcript 2”[tiab] OR “non-coding nuclear-enriched abundant transcript-2”[tiab] OR “non-coding nuclear enriched abundant transcript-2”[tiab] OR “non-coding nuclear enriched abundant transcript 2”[tiab] OR “HCN”[tiab] OR “PRO2853”[tiab] OR “mascRNA”[tiab] OR “LINC00047”[tiab] OR “NCRNA00047”[tiab]) AND (“Colorectal Neoplasms”[MeSH] OR “Colorectal Neoplasm*”[tiab] OR “Neoplasm, Colorectal”[tiab] OR “Neoplasms, Colorectal”[tiab] OR “Colorectal Tumors”[tiab] OR “Colorectal Tumor”[tiab] OR “Tumor, Colorectal”[tiab] OR “Tumors, Colorectal”[tiab] OR “Colorectal Cancer”[tiab] OR “Cancer, Colorectal”[tiab] OR “Cancers, Colorectal”[tiab] OR “Colorectal Cancers”[tiab] OR “Colorectal Carcinoma”[tiab] OR “Carcinoma, Colorectal”[tiab] OR “Carcinomas, Colorectal”[tiab] OR “Colorectal Carcinomas”[tiab] OR “Colonic Neoplasms”[MeSH] OR “Colonic Neoplasm*”[tiab] OR “Neoplasm, Colonic”[tiab] OR “Neoplasms, Colonic”[tiab] OR “Colon Neoplasms”[tiab] OR “Colon Neoplasm”[tiab] OR “Neoplasm, Colon”[tiab] OR “Neoplasms, Colon”[tiab] OR “Cancer of Colon”[tiab] OR “Colon Cancers”[tiab] OR “Colon Cancer”[tiab] OR “Cancer, Colon”[tiab] OR “Cancers, Colon”[tiab] OR “Cancer of the Colon”[tiab] OR “Colonic Cancer”[tiab] OR “Cancer, Colonic”[tiab] OR “Cancers, Colonic”[tiab] OR “Colonic Cancers”[tiab] OR “Colon Adenocarcinoma”[tiab] OR “Adenocarcinoma, Colon”[tiab] OR “Adenocarcinomas, Colon”[tiab] OR “Colon Adenocarcinomas”[tiab] OR “Rectal Neoplasms”[MeSH] OR “Neoplasm, Rectal”[tiab] OR “Rectal Neoplasm*”[tiab] OR “Rectum Neoplasms”[tiab] OR “Neoplasm, Rectum”[tiab] OR “Rectum Neoplasm”[tiab] OR “Rectal Tumors”[tiab] OR “Rectal Tumor”[tiab] OR “Tumor, Rectal”[tiab] OR “Neoplasms, Rectal”[tiab] OR “Cancer of Rectum”[tiab] OR “Rectum Cancers”[tiab] OR “Rectal Cancer”[tiab] OR “Cancer, Rectal”[tiab] OR “Rectal Cancers”[tiab] OR “Rectum Cancer”[tiab] OR “Cancer, Rectum”[tiab] OR “Cancer of the Rectum”[tiab])

ISI:

TS=(“MALAT1” OR “MALAT 1” OR “MALAT-1” OR “metastasis associated lung adenocarcinoma transcript 1” OR “metastasis associated lung adenocarcinoma transcript-1” OR “NEAT2” OR “NEAT 2” OR “NEAT-2” OR “non-coding nuclear-enriched abundant transcript 2” OR “non-coding nuclear-enriched abundant transcript-2” OR “non-coding nuclear enriched abundant transcript-2” OR “non-coding nuclear enriched abundant transcript 2” OR “HCN” OR “PRO2853” OR “mascRNA” OR “LINC00047” OR “NCRNA00047”)

AND

TS=(“Colorectal Neoplasm” OR “Neoplasm, Colorectal” OR “Neoplasms, Colorectal” OR “Colorectal Tumors” OR “Colorectal Tumor” OR “Tumor, Colorectal” OR “Tumors, Colorectal” OR “Colorectal Cancer” OR “Cancer, Colorectal” OR “Cancers, Colorectal” OR “Colorectal Cancers” OR “Colorectal Carcinoma” OR “Carcinoma, Colorectal” OR “Carcinomas, Colorectal” OR “Colorectal Carcinomas” OR “Colonic Neoplasm” OR “Neoplasm, Colonic” OR “Neoplasms, Colonic” OR “Colon Neoplasms” OR “Colon Neoplasm” OR “Neoplasm, Colon” OR “Neoplasms, Colon” OR “Cancer of Colon” OR “Colon Cancers” OR “Colon Cancer” OR “Cancer, Colon” OR “Cancers, Colon” OR “Cancer of the Colon” OR “Colonic Cancer” OR “Cancer, Colonic” OR “Cancers, Colonic” OR “Colonic Cancers” OR “Colon Adenocarcinoma” OR “Adenocarcinoma, Colon” OR “Adenocarcinomas, Colon” OR “Colon Adenocarcinomas” OR “Neoplasm, Rectal” OR “Rectal Neoplasm” OR “Rectum Neoplasms” OR “Neoplasm, Rectum” OR “Rectum Neoplasm” OR “Rectal Tumors” OR “Rectal Tumor” OR “Tumor, Rectal” OR “Neoplasms, Rectal” OR “Cancer of Rectum” OR “Rectum Cancers” OR “Rectal Cancer” OR “Cancer, Rectal” OR “Rectal Cancers” OR “Rectum Cancer” OR “Cancer, Rectum” OR “Cancer of the Rectum”)

Embase:

(“MALAT1”:ti,ab,kw OR “MALAT 1”:ti,ab,kw OR “MALAT-1”:ti,ab,kw OR “metastasis associated lung adenocarcinoma transcript 1”:ti,ab,kw OR “metastasis associated lung adenocarcinoma transcript-1”:ti,ab,kw OR “NEAT2”:ti,ab,kw OR “NEAT 2”:ti,ab,kw OR “NEAT-2”:ti,ab,kw OR “non-coding nuclear-enriched abundant transcript 2”:ti,ab,kw OR “non-coding nuclear-enriched abundant transcript-2”:ti,ab,kw OR “non-coding nuclear enriched abundant transcript-2”:ti,ab,kw OR “non-coding nuclear enriched abundant transcript 2”:ti,ab,kw OR “HCN”:ti,ab,kw OR “PRO2853”:ti,ab,kw OR “mascRNA”:ti,ab,kw OR “LINC00047”:ti,ab,kw OR “NCRNA00047”:ti,ab,kw) AND (“Colorectal Neoplasms”/exp OR “Colorectal Neoplasm*”:ti,ab,kw OR “Neoplasm, Colorectal”:ti,ab,kw OR “Neoplasms, Colorectal”:ti,ab,kw OR “Colorectal Tumors”:ti,ab,kw OR “Colorectal Tumor”:ti,ab,kw OR “Tumor, Colorectal”:ti,ab,kw OR “Tumors, Colorectal”:ti,ab,kw OR “Colorectal Cancer”:ti,ab,kw OR “Cancer, Colorectal”:ti,ab,kw OR “Cancers, Colorectal”:ti,ab,kw OR “Colorectal Cancers”:ti,ab,kw OR “Colorectal Carcinoma”:ti,ab,kw OR “Carcinoma, Colorectal”:ti,ab,kw OR “Carcinomas, Colorectal”:ti,ab,kw OR “Colorectal Carcinomas”:ti,ab,kw OR “Colonic Neoplasms”/exp OR “Colonic Neoplasm*”:ti,ab,kw OR “Neoplasm, Colonic”:ti,ab,kw OR “Neoplasms, Colonic”:ti,ab,kw OR “Colon Neoplasms”:ti,ab,kw OR “Colon Neoplasm”:ti,ab,kw OR “Neoplasm, Colon”:ti,ab,kw OR “Neoplasms, Colon”:ti,ab,kw OR “Cancer of Colon”:ti,ab,kw OR “Colon Cancers”:ti,ab,kw OR “Colon Cancer”:ti,ab,kw OR “Cancer, Colon”:ti,ab,kw OR “Cancers, Colon”:ti,ab,kw OR “Cancer of the Colon”:ti,ab,kw OR “Colonic Cancer”:ti,ab,kw OR “Cancer, Colonic”:ti,ab,kw OR “Cancers, Colonic”:ti,ab,kw OR “Colonic Cancers”:ti,ab,kw OR “Colon Adenocarcinoma”:ti,ab,kw OR “Adenocarcinoma, Colon”:ti,ab,kw OR “Adenocarcinomas, Colon”:ti,ab,kw OR “Colon Adenocarcinomas”:ti,ab,kw OR “Rectal Neoplasms”/exp OR “Neoplasm, Rectal”:ti,ab,kw OR “Rectal Neoplasm*”:ti,ab,kw OR “Rectum Neoplasms”:ti,ab,kw OR “Neoplasm, Rectum”:ti,ab,kw OR “Rectum Neoplasm”:ti,ab,kw OR “Rectal Tumors”:ti,ab,kw OR “Rectal Tumor”:ti,ab,kw OR “Tumor, Rectal”:ti,ab,kw OR “Neoplasms, Rectal”:ti,ab,kw OR “Cancer of Rectum”:ti,ab,kw OR “Rectum Cancers”:ti,ab,kw OR “Rectal Cancer”:ti,ab,kw OR “Cancer, Rectal”:ti,ab,kw OR “Rectal Cancers”:ti,ab,kw OR “Rectum Cancer”:ti,ab,kw OR “Cancer, Rectum”:ti,ab,kw OR “Cancer of the Rectum”:ti,ab,kw)

Scopus:

TITLE-ABS-KEY(“MALAT1” OR “MALAT 1” OR “MALAT-1” OR “metastasis associated lung adenocarcinoma transcript 1” OR “metastasis associated lung adenocarcinoma transcript-1” OR “NEAT2” OR “NEAT 2” OR “NEAT-2” OR “non-coding nuclear-enriched abundant transcript 2” OR “non-coding nuclear-enriched abundant transcript-2” OR “non-coding nuclear enriched abundant transcript-2” OR “non-coding nuclear enriched abundant transcript 2” OR “HCN” OR “PRO2853” OR “mascRNA” OR “LINC00047” OR “NCRNA00047”)

AND

TITLE-ABS-KEY(“Colorectal Neoplasm” OR “Neoplasm, Colorectal” OR “Neoplasms, Colorectal” OR “Colorectal Tumors” OR “Colorectal Tumor” OR “Tumor, Colorectal” OR “Tumors, Colorectal” OR “Colorectal Cancer” OR “Cancer, Colorectal” OR “Cancers, Colorectal” OR “Colorectal Cancers” OR “Colorectal Carcinoma” OR “Carcinoma, Colorectal” OR “Carcinomas, Colorectal” OR “Colorectal Carcinomas” OR “Colonic Neoplasm” OR “Neoplasm, Colonic” OR “Neoplasms, Colonic” OR “Colon Neoplasms” OR “Colon Neoplasm” OR “Neoplasm, Colon” OR “Neoplasms, Colon” OR “Cancer of Colon” OR “Colon Cancers” OR “Colon Cancer” OR “Cancer, Colon” OR “Cancers, Colon” OR “Cancer of the Colon” OR “Colonic Cancer” OR “Cancer, Colonic” OR “Cancers, Colonic” OR “Colonic Cancers” OR “Colon Adenocarcinoma” OR “Adenocarcinoma, Colon” OR “Adenocarcinomas, Colon” OR “Colon Adenocarcinomas” OR “Neoplasm, Rectal” OR “Rectal Neoplasm” OR “Rectum Neoplasms” OR “Neoplasm, Rectum” OR “Rectum Neoplasm” OR “Rectal Tumors” OR “Rectal Tumor” OR “Tumor, Rectal” OR “Neoplasms, Rectal” OR “Cancer of Rectum” OR “Rectum Cancers” OR “Rectal Cancer” OR “Cancer, Rectal” OR “Rectal Cancers” OR “Rectum Cancer” OR “Cancer, Rectum” OR “Cancer of the Rectum”)

**Supplementary Table.** Excluded studies after full text review.

| **ID** | **Title** | **Author, year** | **Reason of exclusion** |
| --- | --- | --- | --- |
| 1 | Long Non-coding RNA Signature for Liver Metastasis of Colorectal Cancers | Liu. F (1), 2021 | No relative diagnostic or prognostic analysis |
| 2 | Association of MALAT1 and PVT1 Variants, Expression Profiles and Target miRNA-101 and miRNA-186 with Colorectal Cancer: Correlation with Epithelial-Mesenchymal Transition | Radwan (2), 2021 | No relative diagnostic or prognostic analysis |
| 3 | Clinical Significance and Function of MALAT1 Gene Expression and the rs619586 Polymorphism in Colorectal Cancer | Ren. G (3), 2022 | Letter to the editor |
| 4 | Long Noncoding RNAs as Prognostic Markers for Colorectal Cancer in Saudi Patients | Siddique. H (4), 2019 | No relative diagnostic or prognostic analysis |
| 5 | Discovery of Long Non-Coding RNA MALAT1 Amplification in Precancerous Colorectal Lesions | Siskova (5), 2022 | No relative diagnostic or prognostic analysis |
| 6 | lncRNAs in Non-Malignant Tissue Have Prognostic Value in Colorectal Cancer | Thiele (6), 2018 | No relative diagnostic or prognostic analysis |
| 7 | Z Probe, An Efficient Tool for Characterizing Long Non-Coding RNA in FFPE Tissues | Tripathi (7), 2018 | No relative diagnostic or prognostic analysis |
| 8 | The contribution of MALAT1 gene rs3200401 and MEG3 gene rs7158663 to the risk of lung, colorectal, gastric and liver cancer | Wang. W (8), 2022 | Meta-analysis |
| 9 | LncRNA MALAT1 promotes colorectal cancer development by sponging miR-363-3p to regulate EZH2 expression | Xie. J (9), 2019 | Full text unavailable |
| 10 | Exosomal MALAT1 sponges miR-26a/26b to promote the invasion and metastasis of colorectal cancer via FUT4 enhanced fucosylation and PI3K/Akt pathway | Xu. J (10), 2020 | Dataset |
| 11 | MALAT1 promotes colorectal cancer cell proliferation/migration/invasion via PRKA kinase anchor protein 9 | Yang. M (11), 2015 | No relative diagnostic or prognostic analysis |
| 12 | MicroRNA Binding Site Polymorphisms of the Long-Chain Noncoding RNA MALAT1 are Associated with Risk and Prognosis of Colorectal Cancer in Chinese Han Population | Yang. Q (12), 2020 | No relative diagnostic or prognostic analysis |
| 13 | Aberrant expression of long noncoding RNAs in colorectal cancer with liver metastasis | Ye. L (13), 2015 | No relative diagnostic or prognostic analysis |
| 14 | WiNTRLINC1/ASCL2/c-Myc Axis Characteristics of Colon Cancer with Differentiated Histology at Young Onset and Essential for Cell Viability | Yokota. K (14), 2019 | No relative diagnostic or prognostic analysis |
| 15 | Association study of genetic variation of lncRNA MALAT1 with carcinogenesis of colorectal cancer | Zhao. K (15), 2018 | No relative diagnostic or prognostic analysis |
| 16 | LncRNA-MALAT1 mediated colorectal cancer cells to 5-fluorouracil resistance by targeting miR-106b-5p | Zhang. P (16), 2021 | Full text unavailable |
| 17 | Long Noncoding RNA MALAT1 Promotes Colorectal Cancer Progression by Acting as a ceRNA of miR-508-5p to Regulate RAB14 Expression | Zhang. C (17), 2020 | Dataset |
| 18 | MALAT1 overexpression promotes the growth of colon cancer by repressing β-catenin degradation | Zheng. X (18), 2020 | No relative diagnostic or prognostic analysis |
| 19 | MALAT1 sponges miR-106b-5p to promote the invasion and metastasis of colorectal cancer via SLAIN2 enhanced microtubules mobility | Zhuang. M (19), 2019 | No relative diagnostic or prognostic analysis |
| 20 | Differential Expression of Decorin in Metastasising Colorectal Carcinoma Is Regulated by miR-200c and Long Non-Coding RNAs | Žlajpah. M (20), 2022 | No relative diagnostic or prognostic analysis |
| 21 | MALAT1 rs664589 Polymorphism Inhibits Binding to miR-194-5p, Contributing to Colorectal Cancer Risk, Growth, and Metastasis | Wu. S (21), 2019 | No relative diagnostic or prognostic analysis |
| 22 | The prognostic value of long noncoding RNAs as biomarkers in early stage colon cancer | Aksoy. S (22), 2017 | Conference abstract |
| 23 | Contribution of Long Non-Coding Rnas (Ccat1, Malat1, and Pandar) in the Pathogenesis of Colorectal Cancer | Al-Ghafari. A. B (23), 2022 | No relative diagnostic or prognostic analysis |
| 24 | Gene expression profile of human colorectal cancer identified NKTR as a biomarker for liver metastasis | Bai. R (24), 2022 | No relative diagnostic or prognostic analysis |
| 25 | LncRNA UCA1, Upregulated in CRC Biopsies and Downregulated in Serum Exosomes, Controls mRNA Expression by RNA-RNA Interactions | Barbagallo. C (25), 2018 | No relative diagnostic or prognostic analysis |
| 26 | Regulation of Colon Cancer Cells Biology by Long Non-Coding RNA Metastasis Associated Lung Adenocarcinoma Transcript 1 (LncRNA MALAT1) via Targeting miR-184 | Bie. J (26), 2022 | Full text unavailable |
| 27 | MALAT1 in Liquid Biopsy: The Diagnostic and Prognostic Promise for Colorectal Cancer and Adenomas? | Cervena. K (27), 2023 | No relative diagnostic or prognostic analysis |
| 28 | Evaluation of MALAT1 promoter DNA methylation patterns in early colorectal lesions and tumors | Chaleshi. V (28), 2019 | No relative diagnostic or prognostic analysis |
| 29 | Detection of p53, malat 1, ki-67 and ß-catenin mRNA expression and its significance in molecular diagnosis of colorectal carcinoma | Chang. J (29), 2008 | Not in English |
| 30 | Stress regulated role of lncRNA Malat1 in colorectal cancer progression and metastasis | Doxtater. K (30), 2020 | Conference abstract |
| 31 | Alterations of non-coding RNA expression and mitochondrial biogenesis in colorectal cancer tissue: Possible crosstalk with macrophage polarization | El-Tahan. R (31), 2021 | No relative diagnostic or prognostic analysis |
| 32 | Metastasis-associated lung adenocarcinoma transcript 1 regulates tumor progression: old wine in a new bottle | He. X. Y (32), 2018 | Letter to the editor |
| 33 | lncRNA MALAT1 regulates the expression level of miR-21 and interferes with the biological behavior of colon cancer cells | Huang. B (33), 2020 | No relative diagnostic or prognostic analysis |
| 34 | Long non-coding RNA MALAT1 promotes tumour growth and metastasis in colorectal cancer through binding to SFPQ and releasing oncogene PTBP2 from SFPQ/PTBP2 complex | Ji. Q (34), 2014 | No relative diagnostic or prognostic analysis |
| 35 | MALAT1 Is Associated Q1 with Poor Response to Oxaliplatin- Based Chemotherapy in Colorectal Cancer Patients and Promotes Chemoresistance through EZH2 | Li. P (35), 2017 | Full text unavailable |
| 36 | Correction: MALAT1 is Associated with Poor Response to Oxaliplatin-based Chemotherapy in Colorectal Cancer Patients and Promotes Chemoresistance through EZH2 | Li. P (36), 2021 | Published Erratum |
| 37 | Differentially expressed long non-coding RNAs and the prognostic potential in colorectal cancer | Li. Q (37), 2016 | No numerical prognostic or diagnostic data |

# References:

1. Liu F, Song ZM, Wang XD, Du SY, Peng N, Zhou JR, et al. Long Non-coding RNA Signature for Liver Metastasis of Colorectal Cancers. Front Cell Dev Biol. 2021;9:707115.

2. Radwan AF, Shaker OG, El-Boghdady NA, Senousy MA. Association of MALAT1 and PVT1 Variants, Expression Profiles and Target miRNA-101 and miRNA-186 with Colorectal Cancer: Correlation with Epithelial-Mesenchymal Transition. Int J Mol Sci. 2021;22(11).

3. Ren GX, Yu WX, Yang LX, Qi SY, Long ZS. Clinical Significance and Function of MALAT1 Gene Expression and the rs619586 Polymorphism in Colorectal Cancer. BIOMEDICAL AND ENVIRONMENTAL SCIENCES. 2022;35(8):768-+.

4. Siddique H, Al-Ghafari A, Choudhry H, AlTurki S, Alshaibi H, Al Doghaither H, et al. Long Noncoding RNAs as Prognostic Markers for Colorectal Cancer in Saudi Patients. Genet Test Mol Biomarkers. 2019;23(8):509-14.

5. Siskova A, Kral J, Drabova J, Cervena K, Tomasova K, Jungwirth J, et al. Discovery of Long Non-Coding RNA MALAT1 Amplification in Precancerous Colorectal Lesions. Int J Mol Sci. 2022;23(14).

6. Thiele JA, Hosek P, Kralovcova E, Ostasov P, Liska V, Bruha J, et al. lncRNAs in Non-Malignant Tissue Have Prognostic Value in Colorectal Cancer. Int J Mol Sci. 2018;19(9).

7. Tripathi MK, Zacheaus C, Doxtater K, Keramatnia F, Gao C, Yallapu MM, et al. Z Probe, An Efficient Tool for Characterizing Long Non-Coding RNA in FFPE Tissues. Noncoding RNA. 2018;4(3).

8. Wang W, Xiong W, Zheng J, Jin Y, Dong L, Feng X, et al. The contribution of MALAT1 gene rs3200401 and MEG3 gene rs7158663 to the risk of lung, colorectal, gastric and liver cancer. Pathol Res Pract. 2022;240:154212.

9. Xie JJ, Li WH, Li X, Ye W, Shao CF. LncRNA MALAT1 promotes colorectal cancer development by sponging miR-363-3p to regulate EZH2 expression. J Biol Regul Homeost Agents. 2019;33(2):331-43.

10. Xu J, Xiao Y, Liu B, Pan S, Liu Q, Shan Y, et al. Exosomal MALAT1 sponges miR-26a/26b to promote the invasion and metastasis of colorectal cancer via FUT4 enhanced fucosylation and PI3K/Akt pathway. J Exp Clin Cancer Res. 2020;39(1):54.

11. Yang MH, Hu ZY, Xu C, Xie LY, Wang XY, Chen SY, et al. MALAT1 promotes colorectal cancer cell proliferation/migration/invasion via PRKA kinase anchor protein 9. Biochim Biophys Acta. 2015;1852(1):166-74.

12. Yang Q, Zheng W, Shen Z, Huang G, Yang G. MicroRNA Binding Site Polymorphisms of the Long-Chain Noncoding RNA MALAT1 are Associated with Risk and Prognosis of Colorectal Cancer in Chinese Han Population. Genet Test Mol Biomarkers. 2020;24(5):239-48.

13. Ye LC, Ren L, Qiu JJ, Zhu DX, Chen T, Chang WJ, et al. Aberrant expression of long noncoding RNAs in colorectal cancer with liver metastasis. TUMOR BIOLOGY. 2015;36(11):8747-54.

14. Yokota K, Tanaka Y, Harada H, Kaida T, Nakamoto S, Soeno T, et al. WiNTRLINC1/ASCL2/c-Myc Axis Characteristics of Colon Cancer with Differentiated Histology at Young Onset and Essential for Cell Viability. Ann Surg Oncol. 2019;26(13):4826-34.

15. Zhao K, Jin S, Wei B, Cao S, Xiong Z. Association study of genetic variation of lncRNA MALAT1 with carcinogenesis of colorectal cancer. Cancer Manag Res. 2018;10:6257-61.

16. Zhang PH, Xu ZG, Yang ML, Yang CJ. LncRNA-MALAT1 mediated colorectal cancer cells to 5-fluorouracil resistance by targeting miR-106b-5p. Chinese Journal of Cancer Prevention and Treatment. 2021;28(12):914-20.

17. Zhang C, Yao K, Zhang J, Wang C, Wang C, Qin C. Long Noncoding RNA MALAT1 Promotes Colorectal Cancer Progression by Acting as a ceRNA of miR-508-5p to Regulate RAB14 Expression. Biomed Res Int. 2020;2020:4157606.

18. Zheng XY, Ren JH, Peng BJ, Ye JL, Wu XC, Zhao WH, et al. MALAT1 overexpression promotes the growth of colon cancer by repressing beta-catenin degradation. CELLULAR SIGNALLING. 2020;73.

19. Zhuang M, Zhao S, Jiang Z, Wang S, Sun P, Quan J, et al. MALAT1 sponges miR-106b-5p to promote the invasion and metastasis of colorectal cancer via SLAIN2 enhanced microtubules mobility. EBioMedicine. 2019;41:286-98.

20. Žlajpah M, Urh K, Grosek J, Zidar N, Boštjančič E. Differential Expression of Decorin in Metastasising Colorectal Carcinoma Is Regulated by miR-200c and Long Non-Coding RNAs. Biomedicines. 2022;10(1).

21. Wu S, Sun H, Wang Y, Yang X, Meng Q, Yang H, et al. MALAT1 rs664589 Polymorphism Inhibits Binding to miR-194-5p, Contributing to Colorectal Cancer Risk, Growth, and Metastasis. Cancer Res. 2019;79(20):5432-41.

22. Aksoy S, Tunca B, Ozturk E, Yilmazlar T, Isik O, Yerci O, et al. The prognostic value of long noncoding RNAs as biomarkers in early stage colon cancer. Colorectal Disease. 2017;19:97.

23. Al-Ghafari AB, Siddiqui HA, Choudhry HM, Al Doghaither HA, Alshaibi HF, Alsufiani HM, et al. Contribution of Long Non-Coding Rnas (Ccat1, Malat1, and Pandar) in the Pathogenesis of Colorectal Cancer. Pakistan Journal of Medical and Health Sciences. 2022;16(2):512-7.

24. Bai R, Shi Z, Li D, Zhou D, Ge WT, Zheng S. Gene expression profile of human colorectal cancer identified NKTR as a biomarker for liver metastasis. Aging (Albany NY). 2022;14(16):6656-67.

25. Barbagallo C, Brex D, Caponnetto A, Cimigliaro M, Scalia M, Magnano A, et al. LncRNA UCA1, Upregulated in CRC Biopsies and Downregulated in Serum Exosomes, Controls mRNA Expression by RNA-RNA Interactions. MOLECULAR THERAPY-NUCLEIC ACIDS. 2018;12:229-41.

26. Bie J, Zeng JR, Wu XX. Regulation of Colon Cancer Cells Biology by Long Non-Coding RNA Metastasis Associated Lung Adenocarcinoma Transcript 1 (LncRNA MALAT1) via Targeting miR-184. JOURNAL OF BIOMATERIALS AND TISSUE ENGINEERING. 2022;12(11):2153-61.

27. Cervena K, Levy M, Siskova A, Jungwirth J, Volarić M, Kral J, et al. MALAT1 in Liquid Biopsy: The Diagnostic and Prognostic Promise for Colorectal Cancer and Adenomas? International Journal of General Medicine. 2023;16:3517-31.

28. Chaleshi V, Irani S, Alebouyeh M, Mirfakhraie R, Aghdaei HA. Evaluation of MALAT1 promoter DNA methylation patterns in early colorectal lesions and tumors. Gastroenterology and Hepatology from Bed to Bench. 2019;12:S58-S65.

29. Chang JL, Li ZG, Wang XY, Yang MH. Detection of p53, malat 1, ki-67 and ß-catenin mRNA expression and its significance in molecular diagnosis of colorectal carcinoma. World Chinese Journal of Digestology. 2008;16(34):3849-54.

30. Doxtater K, Zacheaus C, Sekhri R, Mishra UK, Stiles ZE, Mishra N, et al. Stress regulated role of lncRNA Malat1 in colorectal cancer progression and metastasis. Cancer Research. 2020;80(16 SUPPL).

31. El-Tahan RA, Youssry S, Michel TN, Salman MSK, Kamel MA, Eweda SM. Alterations of non-coding RNA expression and mitochondrial biogenesis in colorectal cancer tissue: Possible crosstalk with macrophage polarization. Gene Reports. 2021;25.

32. He XY, Yan QJ, Kuang GY, Wang YX, Cao PF, Ou CL. Metastasis-associated lung adenocarcinoma transcript 1 regulates tumor progression: old wine in a new bottle. JOURNAL OF THORACIC DISEASE. 2018;10:S1088-S91.

33. Huang B, Guo X, Li Y. lncRNA MALAT1 regulates the expression level of miR-21 and interferes with the biological behavior of colon cancer cells. J buon. 2020;25(2):907-13.

34. Ji Q, Zhang L, Liu X, Zhou L, Wang W, Han Z, et al. Long non-coding RNA MALAT1 promotes tumour growth and metastasis in colorectal cancer through binding to SFPQ and releasing oncogene PTBP2 from SFPQ/PTBP2 complex. Br J Cancer. 2014;111(4):736-48.

35. Li P, Wang C. MALAT1 Is Associated Q1 with Poor Response to Oxaliplatin- Based Chemotherapy in Colorectal Cancer Patients and Promotes Chemoresistance through EZH2. Clinical Chemistry. 2017;63:S6.

36. Li P, Zhang X, Wang H, Wang L, Liu T, Du L, et al. Correction: MALAT1 is Associated with Poor Response to Oxaliplatin-based Chemotherapy in Colorectal Cancer Patients and Promotes Chemoresistance through EZH2. Mol Cancer Ther. 2021;20(2):451.

37. Li Q, Dai Y, Wang F, Hou S. Differentially expressed long non-coding RNAs and the prognostic potential in colorectal cancer. Neoplasma. 2016;63(6):977-83.
